# Building ADMISSION – A research collaborative to transform understanding of multiple long-term conditions for people admitted to hospital

**DOI:** 10.1177/26335565251317940

**Published:** 2025-02-01

**Authors:** Miles D Witham, Victoria Bartle, Sue Bellass, Jonathan G Bunn, Duncan Cartner, Heather J Cordell, Rominique Doal, Felicity Evison, Suzy Gallier, Steve Harris, Susan J Hillman, Ray Holding, Peta Leroux, Tom Marshall, Fiona E Matthews, Paolo Missier, Anand Nair, Mo Osman, Ewan R Pearson, Chris Plummer, Sara Pretorius, Sarah J Richardson, Sian M Robinson, Elizabeth Sapey, Thomas Scharf, Rupal Shah, Marzieh Shahmandi, Mervyn Singer, Jana Suklan, James MS Wason, Rachel Cooper, Avan A Sayer

**Affiliations:** 1 AGE Research Group, Translational and Clinical Research Institute, Faculty of Medical Sciences, 5994 Newcastle University, Newcastle Upon Tyne, UK; 2 NIHR Newcastle Biomedical Research Centre, Newcastle Upon Tyne Hospitals NHS Foundation Trust, 5994 Cumbria, Northumberland, Tyne and Wear NHS Foundation Trust and Newcastle University, Newcastle Upon Tyne, UK; 3 Public Co-Investigator, ADMISSION Research Collaborative, Newcastle Upon Tyne, UK; 4 Department of Sport and Exercise Sciences, 5289 Manchester Metropolitan University, Manchester, UK; 5 4919 University College London Hospitals NHS Foundation Trust, London, UK; 6 Population Health Sciences Institute, Faculty of Medical Sciences, 5994 Newcastle University, Newcastle Upon Tyne, UK; 7 PIONEER Hub, 1724 University of Birmingham, Birmingham, UK.; 8 Data Science Team, Research, Development & Innovation, 1732 University Hospitals Birmingham NHS Foundation Trust, Birmingham, UK; 9 Bloomsbury Institute for Intensive Care Medicine, 4919 University College London, London, UK; 10 Digital Services, 5983 Newcastle Upon Tyne Hospitals NHS Foundation Trust, Newcastle Upon Tyne, UK; 11 Institute of Applied Health Research, 1724 University of Birmingham, Birmingham, UK; 12 School of Computing, 5994 Newcastle University, Newcastle Upon Tyne, UK; 13 Division of Population Health and Genomics, Ninewells Hospital and School of Medicine, 3042 University of Dundee, Dundee, UK; 14 NIHR Newcastle HealthTech Research Centre, 5983 Newcastle Upon Tyne Hospitals NHS Foundation Trust, Newcastle Upon Tyne, UK; 15 Institute of Inflammation and Ageing, 1724 University of Birmingham, Birmingham, UK.; 16 Biostatistics Research Group, Population Health Sciences Institute, 5994 Newcastle University, Newcastle Upon Tyne, UK

**Keywords:** Multiple long-term conditions, multimorbidity, epidemiology, interdisciplinary, mechanisms, hospitalisation

## Abstract

**Background:**

Multiple long-term conditions (MLTCs; commonly referred to as multimorbidity) are highly prevalent among people admitted to hospital and are therefore of critical importance to hospital-based healthcare systems. To date, most research on MLTCs has been conducted in primary care or the general population with comparatively little work undertaken in the hospital setting.

**Purpose:**

To describe the rationale and content of ADMISSION: a four-year UK Research and Innovation and National Institute of Health and Care Research funded interdisciplinary programme that seeks, in partnership with public contributors, to transform care for people living with MLTCs admitted to hospital.

**Research design:**

Based across five UK academic centres, ADMISSION combines expertise in clinical medicine, epidemiology, informatics, computing, biostatistics, social science, genetics and care pathway mapping to examine patterns of conditions, mechanisms, consequences and pathways of care for people with MLTCs admitted to hospital.

**Data collection:**

The programme uses routinely collected electronic health record data from large UK teaching hospitals, population-based cohort data from UK Biobank and routinely collected blood samples from The Scottish Health Research Register and Biobank (SHARE). These approaches are complemented by focused qualitative work exploring the perspectives of healthcare professionals and the lived experience of people with MLTCs admitted to hospital.

**Conclusion:**

ADMISSION will provide the necessary foundations to develop novel ways to prevent and treat MLTCs and their consequences in people admitted to hospital and to improve care systems and the quality of care for this underserved group.

## Introduction

The occurrence of multiple long-term conditions (MLTCs, also commonly referred to as multimorbidity) in a single individual has been described as one of the great challenges facing modern medical research, practice and public health.^1–3^ MLTCs have an enormous impact on people living with these conditions, their families and their communities, and caring for people living with MLTCs constitutes an important part of the work of hospital services across the world. However, comparatively little research has focused on the underpinning causes, consequences, and care of MLTCs in hospital settings. To help address this gap in knowledge, the ADMISSION Research Collaborative was funded through a UK wide Strategic Priorities Fund initiative, hosted collaboratively between UK Research and Innovation (UKRI) and the National Institute for Health and Care Research (NIHR). This paper aims to describe the scope and content of the work being undertaken by the ADMISSION research collaborative and to share examples of innovative methods and practice that the collaborative is generating with the wider MLTCs research community.

## Background to the programme

### The challenge of MLTCs

MLTCs, defined as the coexistence of two or more long-term health conditions,^4^ are common and have been estimated to affect between 15% and 43% of the global adult population, depending on population characteristics, which conditions are included, and which country is studied.^5,6^ This simple definition belies enormous variability in the definition and operationalisation of MLTCs, but also great heterogeneity in the combinations of conditions to be found in any population. MLTCs become more prevalent with increasing age, such that more than half of people aged 65 and over are estimated to be living with MLTCs,^7^ and the prevalence is predicted to increase over the next 15 to 20 years.^8,9^ MLTCs do not affect only older people; many younger and middle-aged people also live with MLTCs. Health outcomes are worse for people with MLTCs than for people with single conditions or no conditions: people living with MLTCs have higher mortality rates and higher symptom and treatment burdens.^10–13^ People living with MLTCs are also underserved by current research, being frequently excluded from clinical trials investigating single conditions. This exclusion is critical in explaining why existing guidelines for single conditions are not designed for people living with MLTCs; when guidelines are applied to individuals with MLTCs they frequently lead to conflicting advice^14^ and a high burden of treatment and self-care.^15^

### MLTCs and people admitted to hospital

Most existing research on MLTCs has been conducted in population-based cohorts or within primary care. People admitted to hospital commonly have two or more long-term conditions, but current hospital systems are designed around a paradigm of acute illnesses affecting a single organ system, rather than chronic conditions affecting multiple organ systems.^16^ The majority of hospital physical infrastructure, structures and processes of care, and team expertise and organisation are predicated on this single condition paradigm. Perhaps as a result, people admitted to hospital with MLTCs have been found to report lower satisfaction with care and longer lengths of stay.^11,13,17^

### Gaps in knowledge

Compared to our knowledge of single conditions, our knowledge of MLTCs across a broad range of areas is lacking, from aetiology and risk factors through to treatment and health service delivery. To address key questions in MLTCs research, including how to design and deliver more effective prevention strategies, treatments and services, we need to improve our fundamental knowledge about MLTCs in a range of settings. Building on the existing work of expert groups and public engagement groups,^1–5,16,18^ the following key questions relevant to hospital care for people living with MLTCs are highlighted in Box 1, and the paradigm underpinning the work of the ADMISSION collaborative is shown in Supplemental Figure 1.

**Box 1.** Key questions in MLTCs research with a focus on hospital care.

• What are the prevalence, burden and consequences of MLTCs among people admitted to hospital? • What are the common patterns of MLTCs, including, but not limited to clusters of conditions? • What is the impact of MLTCs on outcomes after hospital admission?
• How do we prevent or minimise inequalities in the development, progression and treatment of MLTCs? • What are the risk factors for the development and progression of MLTCs, including potential drivers of inequality including age, sex, deprivation and ethnicity? • What is the lived experience of people admitted to hospital with MLTCs and how might their biographical life experience influence this?
• How can we redesign hospital to better meet the needs of people admitted with MLTCs? • What factors influence the risk of hospital admission with MLTCs? • What are the care pathways that people with MLTCs follow once admitted to hospital? • How do clinicians make decisions about the care of people with MLTCs admitted to hospital?
• What are the biological mechanisms that underpin the development, and progression of MLTCs, and which contribute to hospitalisation and subsequent adverse outcomes for people with MLTCs? • How can we develop interventions that prevent or mitigate the development, progression and adverse outcomes of MLTCs?
• How can we train and support a workforce that is best equipped to deliver high-quality care for people living with MLTCs?

For all these areas, it is essential to understand the role of inequality in the patterns, causes, consequences and care of MLTCs, including differences due to age, sex, deprivation and ethnicity.

### Strategic Priorities Fund call, design and development

The funding call through which the ADMISSION research collaborative is supported was announced by UK Research and Innovation (UKRI) in early 2020 with funding decisions made in November 2020. ADMISSION was granted four years of funding with a start date of March 2021. In order to deliver the breadth of work envisaged, an interdisciplinary collaborative was assembled, with expertise from patient and public members, clinical medicine, epidemiology, clinical informatics, computing, biostatistics, social science, genetics and care pathway mapping. A range of clinical specialities are represented including primary care, cardiovascular medicine, respiratory medicine, geriatric medicine and critical care.

## Patient and Public Involvement and Engagement (PPIE)

ADMISSION builds on existing public engagement and priority setting work on long-term conditions led by the James Lind Alliance.^18^ A series of workshops with people living with MLTCs and their carers were held during the development of the funding bid, and included men and women of different ages, ethnic background, areas of the country and levels of deprivation, and with lived experiences of different combinations of long-term conditions. We sought advice and feedback on the aims, content and presentation of the bid. Significant changes were made in the light of feedback from the workshops, including adding a dedicated strand of qualitative work to more adequately capture lived experience and adding a package of work focused on care pathways within hospital. During the evolution of the bid, we included two contributors to the workshops as co-applicants, who subsequently became co-investigators.

Both ADMISSION public co-investigators attend the programme management group meetings held once a month and play an active role in reviewing progress of work packages, providing oversight of the programme and representing ADMISSION at external events. We also established a diverse, engaged Patient Advisory Group (PAG) at the start of the programme, whose advice and perspectives inform all of the programme’s activities from planning to dissemination. This group meets quarterly with support from a designated PPIE coordinator, academic lead and other members of the ADMISSION team to train and support the PAG, and also provides ad-hoc contribution to specific tasks including document review and interview piloting. New ADMISSION collaborative members receive an introduction to PPIE as part of their induction programme, and regular evaluation of PPIE activity forms part of the work of the PAG and the ADMISSION team.

## Answering key questions in MLTCs research

### Overview of data sources and methods

**Figure 1.**
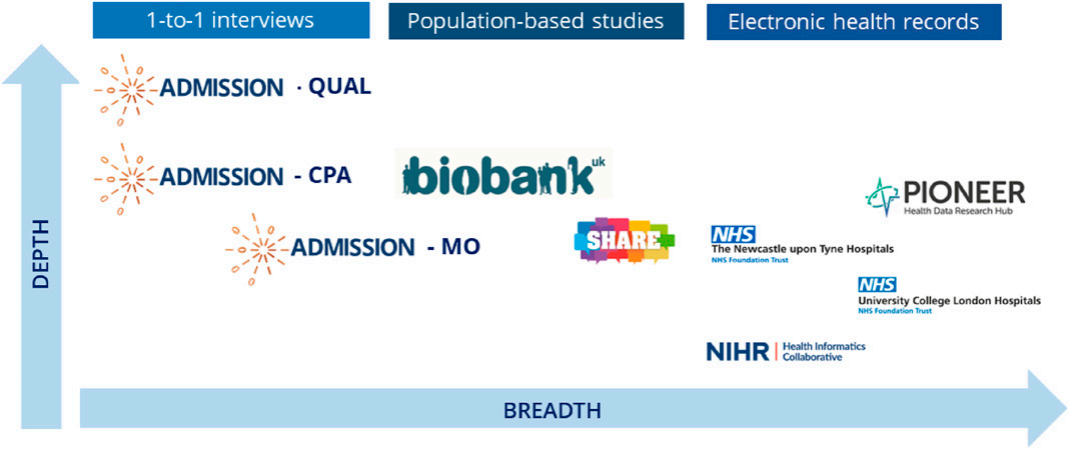
Data sources used in the ADMISSION programme. QUAL: Qualitative study. CPA: Care Pathway Analysis. MO: Mass Observation.

The overarching vision of the ADMISSION Research Collaborative is to transform understanding for people with MLTCs admitted to hospital in the UK National Health Service (NHS) and across the world. The ADMISSION programme uses multiple methods and data sources, ranging from detailed qualitative work to understand the in-depth lived experience of people admitted to hospital with MLTCs, population-based cohorts including UK Biobank for epidemiology and genetic epidemiology, and health informatics approaches using routinely collected electronic health record data from hospitals. Figure 1 shows the complementary range of data sources used in ADMISSION, and how using these data sources enable a balance between breadth (size of study populations) and depth of information (granularity of detail available on each individual) – an approach supported by our Patient Advisory Group.

## Defining and operationalising MLTCs

### Defining a list of long-term conditions for use in ADMISSION

A key initiative within ADMISSION has been to define a standard list of conditions to include when studying MLTCs in the hospital setting. Comparison across studies of MLTCs is currently hampered by the large number of sets of conditions and the variability in how these conditions are operationalised (e.g. ICD-10 codes, SNOMED CT or Read codes);^19^ different sets of conditions result in differences in estimates of prevalence and associations, and some important types of long-term condition (for instance mental health conditions) are often excluded from standard lists. Building on a Delphi consensus exercise published in 2022,^20,21^ we have defined and operationalised 60 conditions to include in ADMISSION analyses.^22^ ICD-10 code lists for these conditions have been derived by drawing on existing published code lists^23^ and working in close collaboration with clinical experts, our patient advisory group, and a hospital-based clinical coding team. This rigorous, standardised and transparent approach to the definition and operationalisation of MLTCs within the ADMISSION programme will make it easier to compare findings within the programme and also provides a template and resource for others researching MLTCs in hospitals. (Box 2)

**Box 2.** Key output: Establishing principles for the identification of a list of LTCs that can be used to define MLTCs in a UK hospital setting.^22^

- We adapted published principles^20^ to guide selection of long-term conditions for inclusion in studies of MLTCs in hospitals
- We applied these principles to two lists of conditions: 59 conditions identified via an international Delphi consensus study and 100 most prevalent HES
- This led to the identification of 60 conditions which we recommend are used when defining MLTCs in hospitalised patients
- This list of 60 conditions comprises 56 of the conditions from the Delphi list and an additional 4 from the HES list
- Two conditions were modified to clarify their definition and improve relevance to patients admitted to hospital
- ICD-10 codes were identified for each of the 60 conditions to ensure consistency in their operationalisation across datasets
- The final list of 60 conditions has been published^22^ with accompanying ICD-10 code lists, which will be shared on a public repository
- This work provides an agreed set of conditions that will underpin future analyses within the ADMISSION collaborative but will also enable others to undertake comparative MLTC research in different studies

## Understanding patterns of MLTCs

ADMISSION seeks to understand patterns of MLTCs at both the population level and at the level of individual patients, using a range of techniques drawing on findings from previous work by ADMISSION collaborators^24^ and other relevant developments in the field. We seek to describe the epidemiology of MLTCs including prevalence of individual conditions recorded during hospital stay, prevalence of MLTCs, through to describing patterns of conditions affecting individuals – including how conditions cluster together at the level of individual patients. To do this, ADMISSION uses data from routinely collected electronic health records, and also uses data from large population-based studies, including UK Biobank which contains genetic and phenotypic data on ∼500,000 individuals.^25^ Interest in clustering remains high, both because clustering may be a way to reduce the number of combinations of LTCs to a tractable number of groups, but also as a way to gain insights into underlying common mechanisms and risk factors.^26^ However, there are some limitations to using clustering. Although some clusters (e.g. for cardiometabolic diseases) appear in most if not all analyses conducted to date, other clusters identified in any one dataset have not been reproducible in other datasets. Secondly, clusters are different for different age groups, and clusters may not be stable over time within a cohort of people living with MLTCs.^27^ Thirdly, clusters may not associate with different outcomes (for example death or risk of hospital admission) in a consistent way. As a result, challenges exist in predicting or generalising the impact of these clusters on individuals moving through healthcare systems. Deploying a range of analyses, from simple counts of conditions through to statistically derived clusters, enables a flexible, responsive and pragmatic approach in this rapidly evolving field, which de-risks the work and maximises the opportunities to produce tractable, actionable research insights on patterns of MLTCs.^28^

## Understanding inequalities in MLTCs

There are stark inequalities in MLTCs at the population level, thus it is of key public health importance to understand how inequalities manifest in hospital settings and work to mitigate them.^2,29^ A key thread running through all work in ADMISSION is to understand how socio-demographic factors, including age, sex, ethnicity and deprivation, intersect and contribute to inequalities in the occurrence and consequences of MLTCs in people admitted to the hospital. Analyses of patterns of MLTCs (from counts to clustering) and their consequences (for instance length of stay, readmission, death) take associations with these different drivers of inequality into consideration, as do analyses of care pathways. For ethnicity, data from electronic health records uses the available NHS ethnicity categories; deprivation is described via the index of multiple deprivation (IMD) obtained from postcodes. Furthermore, qualitative work seeks to represent a range of ages, sex, deprivation and ethnicity in describing both care pathways and the lived experience of MLTCs.

### Describing care pathways for people with multiple long-term conditions

ADMISSION takes a multifaceted approach to understanding the pathways that people living with MLTCs follow as they move through their hospital journey, how antecedent events influence these pathways and the consequences of following particular pathways. ADMISSION uses routinely-collected hospital data as outlined above to describe pathways that individuals follow as they move from hospital admission to discharge.^30^ Moves between departments and wards form the framework for these analyses, augmented by more granular information describing events that occur during a hospital stay. Pathways followed are diverse and complex, and a key aspect of the work is to develop methods to make analysis of such pathways tractable, using techniques including Markov state modelling and machine-learning driven analysis of pathway clustering. These techniques then enable analysis of how sociodemographic factors and patterns of conditions relate to care pathways. Box 3 highlights some of our initial work on this topic.

**Box 3.** Key output: Describing care pathways through hospital stay via transition state probabilities.^30^

- We used routinely-collected data from admissions due to COPD exacerbation held by the HDRUK acute data hub (PIONEER)
- We developed Markov state transition models to depict moves between different places of care (wards or other departments) during hospital stay
- We compared these pathways to a consensus optimal care pathway, and evaluated how closely different subgroups of patients (e.g. those with MLTCs vs those without MLTCs) followed these optimal pathways
- Having 4 or more long-term conditions was associated with higher mortality and longer length of stay; Older patients (aged 70 or over) were less likely to follow optimal pathways but patients with MLTCs were no less likely to follow optimal care pathways
- This work provides a template for future analyses using much larger datasets encompassing a wide range of patients admitted to hospital with and without MLTCs

These analyses are complemented by qualitative enquiry into how people with MLTCs experience their stay in hospital, what constitutes good care, and where care did not meet their needs. This forms part of the ADMISSION qualitative work described below, which sits alongside work to understand care pathways for people with MLTCs admitted to hospital from the perspective of health care professionals. Care pathway mapping is a technique widely used to understand how healthcare is organised and is a critical tool in understanding where a new diagnostic test or therapeutic intervention should be placed within existing healthcare structures.^31^ Care pathway mapping uses analysis of guidelines, standards and protocol reviews, interviews with healthcare professionals, surveys and clinical audits. It seeks to understand formal and informal care pathways, how clinicians make decisions, and how healthcare systems work in practice.^32^ Such techniques have rarely been applied to MLTCs research. ADMISSION is partnering with the NIHR Newcastle HealthTech Research Collaborative (HRC), which has particular expertise in this area. The work of this project (ADMISSION-Care Pathway Analysis; ADMISSION-CPA) involves interviewing a range of clinicians and hospital managers to delineate decision-making and care pathways for people admitted to hospital with MLTCs, with a particular focus on decisions early in the hospital stay.

### Qualitative work in ADMISSION – understanding the lived experience of people with MLTCs

ADMISSION-QUAL is a qualitative study nested within ADMISSION. It seeks to understand the lived experience of people with MLTCs admitted to hospital, both in terms of living with their conditions and also how they experience care during their hospital stay. This work will address key gaps in the current qualitative literature identified by a scoping review undertaken by members of the ADMISSION team (Box 4).^33^ People aged 18 and over with two or more self-reported long-term conditions (including, but not limited to the 60 conditions we discuss above) who have been a hospital inpatient within the last six months are eligible; participants undergo a baseline semi-structured interview with future plans for approximately half the sample to undergo additional two interviews at a later date. Participants are being purposively selected to ensure diversity of age, sex, socioeconomic group and health conditions. They are recruited from three NHS sites in England (Newcastle, Gateshead and Salford) and also via a range of non-NHS recruitment channels. Mixed-methods analysis techniques will be employed using a convergent parallel design to bring together the qualitative findings with ongoing quantitative work on inequalities, care pathways and patterns of conditions.

**Box 4.** Key outputs: Scoping review of qualitative work examining experiences of hospital care for MLTCs.^33^

- We conducted a scoping review to understand what qualitative research had been done previously on this topic
- We included 54 papers, examining views of patients, carers and healthcare professionals
- Key findings included a lack of person-centred care, a time-pressured system with rushed transitions of care, poor integration between services, and a lack of confidence in healthcare professionals on how to treat conditions outwith their specialist area
- Many studies are not explicit in studying people living with MLTCs
- Little work has been done on inequalities, younger people living with MLTCs, or people living with mental health conditions as part of their MLTCs

ADMISSION-QUAL is complemented by ADMISSION-MO, a study conducted with the Mass Observation Project,^34^ a social history archive which captures and preserves accounts of everyday life in Britain written by members of the public. In autumn 2023, members of the ADMISSION team commissioned the Mass Observation Project to send a series of questions to their panel of writers to elicit narratives of MLTCs experiences. Over 140 accounts have been returned to date, covering various aspects of the lived experience of MLTCs, including awareness and understanding, everyday living, health and social care and experiences of ageing with MLTCs across the life course. Both ADMISSION-QUAL and ADMISSION-MO were prepared with input from the Patient Advisory Group, members of whom have contributed to study design, topic guide content and study documentation, coding, analysis, interpretation and dissemination to ensure their relevance for people living with MLTCs.

### Understanding and measuring quality of stay

Focusing solely on metrics such as place of stay and length of stay is likely to miss important aspects of care for people hospitalised with MLTCs. A broader range of quality measures is therefore desirable, but tools do not currently exist to measure quality of care in hospital for this patient group. Qualitative work from ADMISSION-QUAL and linked projects are starting to highlight which aspects of care quality are important for people with MLTCs admitted to hospital, and this is complemented by work combining existing patient-related outcome measures and work on metrics that could be derived from electronic health records using frameworks such as the Institute of Medicine six dimensions of quality.^35^ The aim of this work is to provide a comprehensive, multidimensional understanding of quality of stay for people with MLTCs admitted to hospital.

## Exploring mechanisms underlying multiple long-term conditions

Understanding the shared underpinning biological mechanisms for MLTCs is critical both to understand whether any observed clusters of conditions share common biological mechanisms, and to suggest interventions that could prevent or treat multiple conditions at the same time.^3^ ADMISSION is using a range of genetic epidemiology techniques in conjunction with phenotypic confirmatory studies to address this challenge. By borrowing established techniques in genetic epidemiology and marrying these to a range of ways to characterise patterns of MLTCs, we aim to make progress in unravelling genetic signals in the epidemiology of MLTCs.

### Genetic epidemiology approaches

Genome-wide association studies (GWAS) identify genetic factors associated - potentially causally - with complex diseases and disease-related traits such as diabetes, chronic kidney disease, inflammatory bowel disease, depression, dementia, rheumatoid arthritis and hypertension.^36^ The output from a GWAS is a list of genetic variants that correlate with the disease under study and their genomic locations; further follow-up work is then required to identify potential candidate genes, biological mechanisms and pathways that are implicated by the GWAS findings.

While large-scale GWAS of many of the conditions commonly seen in the context of MLTCs have previously been carried out, there remains a gap in terms of GWAS of combinations of diseases contributing to MLTCs associated with hospitalisation, which ADMISSION seeks to fill. We also plan to carry out GWAS of novel outcomes related to MLTCs and GWAS of latent factors underpinning MLTCs. All of these analyses can be carried out using UK Biobank genetic and phenotypic resources.^25^

Results from previously conducted GWAS of relevant conditions can also be used in novel ways to investigate mechanisms underpinning MLTCs. One approach that ADMISSION is exploring is to cluster individuals based on their polygenic risk scores (PRS) for the relevant conditions. A PRS sums up the effects of all risk variants present in an individual and quantifies the individual’s genetic risk profile as a score. Examining the phenotypic profile of the clusters derived on the basis of these scores represents an alternative way to investigate commonality between the mechanisms underlying conditions, in comparison to clustering based on the conditions themselves. Another approach is to use methods and software for genetic correlation analysis^37^ applied to conditions chosen because they contribute to MLTCs. Such analyses have already provided evidence for global genetic correlation between many pairs of conditions; an arguably more interesting endeavour will be to investigate *local* genetic correlations (localised to a small region of the genome), allowing us to interrogate the genes in the implicated regions and explore possible underpinning biological mechanisms.

### Phenotypic confirmation

Once promising candidate mechanisms are identified via genetic epidemiology, we plan targeted confirmatory studies using stored samples from the SHARE Scotland Biobank.^38^ This biobank contains spare blood samples left over from routine clinical testing, including during hospital stay for approximately 30,000 people admitted to hospital in Scotland with linkage to routinely collected clinical data. Biomarkers relevant to the underlying mechanism (e.g. for inflammation, senescence, DNA damage, oxidative stress) will be analysed from people with MLTCs admitted to hospital and compared across different patterns of conditions and inequalities to provide confirmation of candidate associations.

## Delivering better data for MLTCs research

### Use of electronic health record data in ADMISSION

ADMISSION uses data from three hospital groups: a) the University Hospitals Birmingham NHS Foundation Trust (UHB), b) the Newcastle upon Tyne Hospitals NHS Foundation Trust (NuTH), and c) the University College London Hospital NHS Foundation Trust (UCLH). These are all large NHS Trusts, providing secondary care services to populations of approximately one million people, and tertiary care services to populations of up to three million people. Each Trust covers multiple hospitals and other healthcare facilities such as community hubs. The included hospitals cover geographically distinct regions in the north, middle and south of England, with each Trust covering a mixture of affluent and deprived populations. The ethnic makeup of served populations differs between Trusts, with high numbers of people of South Asian origin served by UHB and a diverse range of ethnicities served by UCLH. ADMISSION also uses data from the Critical Care Health Informatics Collaborative^39^ - by including a source of detailed critical care data, we are able both to analyse the impact of MLTCs in this under-researched area, and to understand how critical care pathways for people living with MLTCs interface with the broader set of pathways of hospital care.

### Ethics and governance

Approval for the use of routine clinical data in ADMISSION is granted via multiple different routes. Data from PIONEER, the Health Data Research UK (HDR UK) in Acute Care using data from University Hospitals Birmingham NHS Foundation Trust, are approved via umbrella ethics and Confidentiality Advisory Group (CAG) approvals (East Midlands–Derby Research Ethics Committee reference: 20/EM/0158 and CAG reference: 20/CAG/0084).^40^ For data from Newcastle upon Tyne Hospitals NHS Foundation Trust, similar umbrella governance approvals are used (East Midlands–Derby Research Ethics Committee reference: 20/EM/0186 and CAG reference: 21/CAG/0003); use of critical care data from UCLH is permitted under the Critical Care Health Informatics Collaborative generic approvals (London Research Ethics Committee 14/LO/1031 and CAG reference 14/CAG/1001).^39^ Individual participant consent for data use is not required under any of these approvals. The ADMISSION-QUAL qualitative study is approved by the Wales Research Ethics Committee (reference 23/WA/0045) and the ADMISSION-CPA study is approved by Newcastle University ethics committee (reference 39496/2023).

### Compatibility and alignment

An early decision was taken to use a federated approach to data in ADMISSION – rather than attempting to export routine data across organisational boundaries (with the governance challenges that this raises), we sought to develop aligned datasets within each hospital Trust that could support the same analysis done in the same way using the same variables. Initial work highlighted considerable differences between the data sources in terms of what data were available from each hospital Trust, how these data were coded and stored, and how a single data ask was interpreted at each site. Our initial efforts on alignment have focussed on understanding these differences, particularly with reference to how diagnosis (ICD-10 and SNOMED CT) codes are used at each site and how sociodemographic factors are recorded. In conjunction with our work on developing a standard list of conditions to define MLTCs for ADMISSION^22^ we have developed standard coding lists which are now interpreted in the same way across sites. At present, not all sites are using a data standard such as the Observational Medical Outcomes Partnership (OMOP) common data model; this remains an option for future development.

### Moving beyond ICD-10 codes and standard demographic information

A strength of hospital electronic health records is the rich variety of longitudinal data they contain, and ADMISSION is seeking ways to improve diagnostic coding by using these data to add to ICD-10 and SNOMED CT diagnostic codes. Examples include using natural language processing techniques^41^ to extract diagnoses that are poorly coded in ICD-10 (for example sarcopenia) and using medication lists and laboratory data to identify diagnoses that may not be well recorded in hospital (for example hypothyroidism and chronic kidney disease). Linking administrative data (e.g. local and national government) holds out the promise of more nuanced measures of drivers of inequality. For example, we currently use the index of multiple deprivation use as an indicator of deprivation as this can be derived from post codes that are routinely captured in electronic health records and it is a robust and widely-used measure, facilitating comparison across studies.^42,43^ However, IMD may not accurately reflect all relevant aspects of an individual’s socioeconomic position. These additional data links require further evolution of both the technical and governance environments within which ADMISSION operates but they remain an aspiration for the programme.

## Research capacity development

Delivering interdisciplinary research in the field of MLTCs requires a highly skilled workforce, with particular expertise in using large routinely-collected datasets and with the ability to understand and work with colleagues across disciplinary boundaries, for example social science, epidemiology and data science. A growing number of early career researchers (ECRs) have been employed either directly or indirectly (for example on methodology internships) by the ADMISSION programme, engaged in data analysis and curation, qualitative work, care pathway mapping and genetic epidemiology. ADMISSION also provides opportunities for other ECRs based at participating institutions to contribute to the work of the programme and to interact with and learn from colleagues across multiple disciplines through regular work package meetings, seminars and our annual MLTCs symposium. Regular interaction with the PAG ensures that all ADMISSION researchers including ECRs are supported and trained in effective, inclusive PPIE as part of their work and development. ADMISSION has taken an innovative approach to designing posts for data science, recruiting colleagues and supporting students with different professional backgrounds to equip researchers with the skills that will be critical to future research.

To support ECR across different UKRI-NIHR Strategic Priorities Fund (SPF) led initiatives, ADMISSION contributes to a series of communities of practice, including in qualitative research (led from ADMISSION), statistical methods, clinical context and pathways, and patient and public involvement. These communities of practice enable sharing of methods, pooling of resources, facilitate interdisciplinary learning and provide peer support for early- to mid-career researchers within the MLTCs research community.

## Delivering the work: ADMISSION organisation and governance

**Figure 2.**
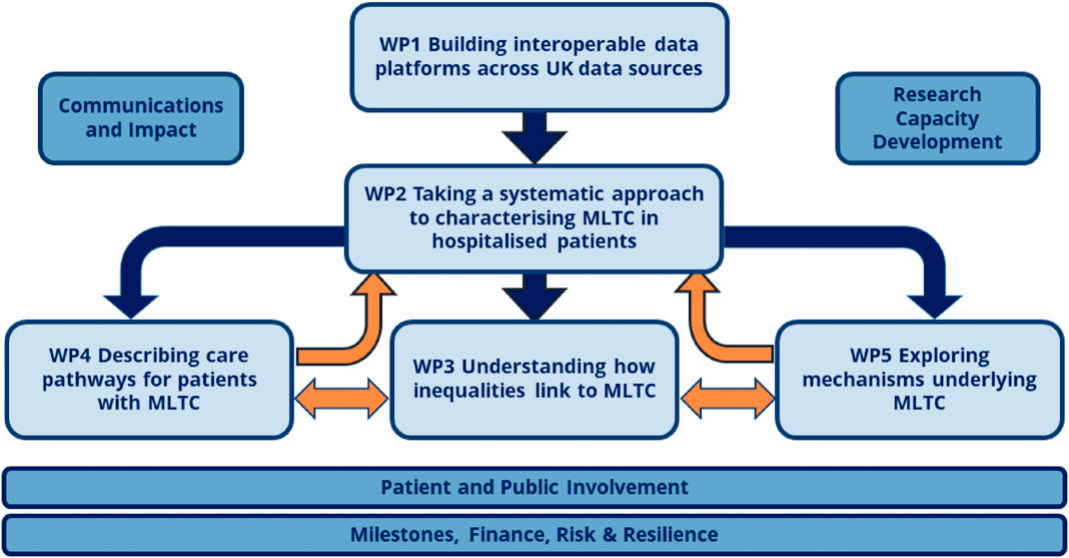
Work package organisation for the ADMISSION programme.

The ADMISSION collaborative comprises 17 co-investigators from five UK academic institutions – the Universities of Newcastle (the lead institution), Birmingham, Dundee, Manchester Metropolitan and UCL. The activity of the programme is organised into five scientific work packages, together with four cross-cutting work packages, as shown in Figure 2. The four supporting work packages are i) Milestones, Finance, Risk and Resilience (to provide overarching governance support); ii) Communications and Impact (to coordinate dissemination of outputs, engagement with a broad range of stakeholders, and planning of externally-facing events such as the annual UK-wide MLTCs symposium); iii) Research Capacity Development (focused on training and development of early-career researchers in the field of MLTCs research); and iv) Patient and Public Involvement and Engagement, as described above.

Monthly Programme Management Group meetings are held to direct the overall work of the programme, with monthly work package meetings to address operational issues. An international External Advisory Group meets annually to provide strategic oversight and guidance. Annual written reports to the funder are accompanied by regular meetings via a Strategic Priorities Fund oversight group to review progress of the programme against agreed milestones. Although work packages are shown as discrete entities, ADMISSION benefits from extensive cross-representation across work packages, which work collaboratively to plan and deliver specific projects within the overall programme.

## Discussion

### ADMISSION in context

Recognition of the importance of MLTCs and the need for more research on MLTCs has catalysed several funding initiatives within the UK and more widely.^44^ ADMISSION is one of six programmes within the UKRI-NIHR Strategic Priorities Fund (SPF) ‘Tackling multimorbidity at scale: understanding disease clusters, determinants and biological pathways’ initiative; other programmes have a focus on MLTCs and pregnancy (MuM-PreDiCT), genetic epidemiology (GEMINI), fibrosis (DEMISTIFI), patterns and mechanisms (MMTRC) and the interface between physical and mental health (LINC).^45^ Other funding streams are supporting work on artificial intelligence in MLTCs research. ADMISSION therefore forms part of an increasingly complex landscape of funded programmes of MLTCs research and in recognition of this, NIHR have recently launched a cross-NIHR collaborative in MLTCs to join up different relevant aspects of NIHR research infrastructure to coordinate MLTCs research and better address the research challenges it presents.

### Interdisciplinary approach

Taking an integrative, interdisciplinary approach, and working in partnership with patients and the public, enables ADMISSION to address multiple facets of MLTCs research. The ability to synthesise findings from different areas of enquiry, using different methods, provides a more complete picture of the causes and consequences of MLTCs in the hospital setting. Our ability to combine qualitative enquiry into the lived experience of people with MLTCs admitted to hospital and information on care pathways as perceived by clinicians, combined with quantitative methods to understand pathways and inequalities is but one example; the research methods are complementary, and each provides new insights for other disciplines to build on in a reciprocal way.

### Capacity development

Supporting researchers to develop the requisite skills to work across disciplinary boundaries, who understand the complexity and nuance of MLTCs as a field, is a key area of activity for ADMISSION. We have created novel posts to enable cross-disciplinary training of clinical staff in data science and epidemiology, created internships and studentships, provided opportunities for data science and statistician colleagues to work closely and learn from clinical colleagues, and leveraged our expertise to support doctoral training in quantitative and qualitative methodologies. ADMISSION contributes to a range of communities of practice to share knowledge and skills in MLTCs research. We have also held annual symposia to bring together the UK MLTCs research community to discuss topical areas of MLTCs research. Early-career researchers and public contributors are included in these events, which provide opportunities both to learn and to showcase their achievements to the wider community.

## Challenges

MLTCs research is a relatively new and rapidly evolving field. A challenge for ADMISSION, and other related programmes, is keeping abreast of developments in both methodology and results. Outputs are often published in a wide range of discipline-specific journals, and as yet there are few events that provide a forum for researchers to meet in the way that is common for single-condition research fields. Developing ways to bring together diverse sets of researchers to exchange knowledge and skills efficiently whilst remaining connected to the broader healthcare research community needs to be a priority for the MLTCs research community.

The use of routinely collected data holds great promise for advancing MLTCs research, but navigating governance processes, obtaining access to such data, understanding its characteristics and limitations,^46^ and aligning approaches across data sources and studies all remain significant challenges to its effective use.^47^ National UK initiatives such as the creation of sub-national Secure Data Environments^48^ to enable pooling and linkage of health and social care data from multiple organisations will help to address these issues but the cost, time and complexity of using routinely collected data require further work, particularly in aligning data and extraction processes across different data providers.

Our experience in ADMISSION has highlighted that MLTCs research requires a research workforce with the appropriate skills, knowledge, training and relationships; interdisciplinary working and effective working with patients and the public are essential to successfully meet the complex challenges that MLTCs pose.^49^ Finding, developing and retaining people with the interest and skills to pursue MLTCs research is crucial to making progress. For some skills (particularly in data science), demand outstrips supply, and salaries in the private sector make retention in academia challenging. New ways to train researchers with interdisciplinary skills, innovative posts, and new incentives and ways of working will all be required to meet these challenges.

## Conclusion

Transforming care for people admitted to hospital with MLTCs is crucial if our current healthcare systems are to survive.^16^ Meeting this challenge will require major foundational research to better understand mechanisms, patterns, causes and consequences of MLTCs, and it is this foundational work that the ADMISSION programme seeks to deliver. Major research efforts are underway both within the UK and globally to tackle MLTCs, and these efforts will need to deliver radically new ways to think about prevention, treatment and healthcare delivery for people living with MLTCs. Designing these new approaches will rest heavily on scientific insights from ADMISSION and similar programmes, and such insights are necessary if we are to break out of the current paradigm of considering one disease at a time in our approach to research, treatment and care.

## Supplemental Material

Supplemental Material - Building ADMISSION – A research collaborative to transform understanding of multiple long-term conditions for people admitted to hospital Supplemental Material for Building ADMISSION – A research collaborative to transform understanding of multiple long-term conditions for people admitted to hospital by Miles D Witham, Victoria Bartle, Sue Bellass, Jonathan G Bunn, Duncan Cartner, Heather J Cordell, Rominique Doal, Felicity Evison, Suzy Gallier, Steve Harris, Susan J Hillman, Ray Holding, Peta Leroux, Tom Marshall, Fiona E Matthews, Paolo Missier, Anand Nair, Mo Osman, Ewan R Pearson, Chris Plummer, Sara Pretorius, Sarah J Richardson, Sian M Robinson, Elizabeth Sapey, Thomas Scharf, Rupal Shah, Marzieh Shahmandi, Mervyn Singer, Jana Suklan, James MS Wason, Rachel Cooper, Avan A Sayer in Journal of Multimorbidity and Comorbidity
