# Supplementary figures and images for: Building ADMISSION – A research collaborative to transform understanding of multiple long-term conditions for people admitted to hospital
Source: J Multimorb Comorb. 2025 Feb 1;15:26335565251317940. doi: 10.1177/26335565251317940 (PMC11787725; doi:10.1177/26335565251317940)

## Supplementary material: ADMISSION paradigm workflow

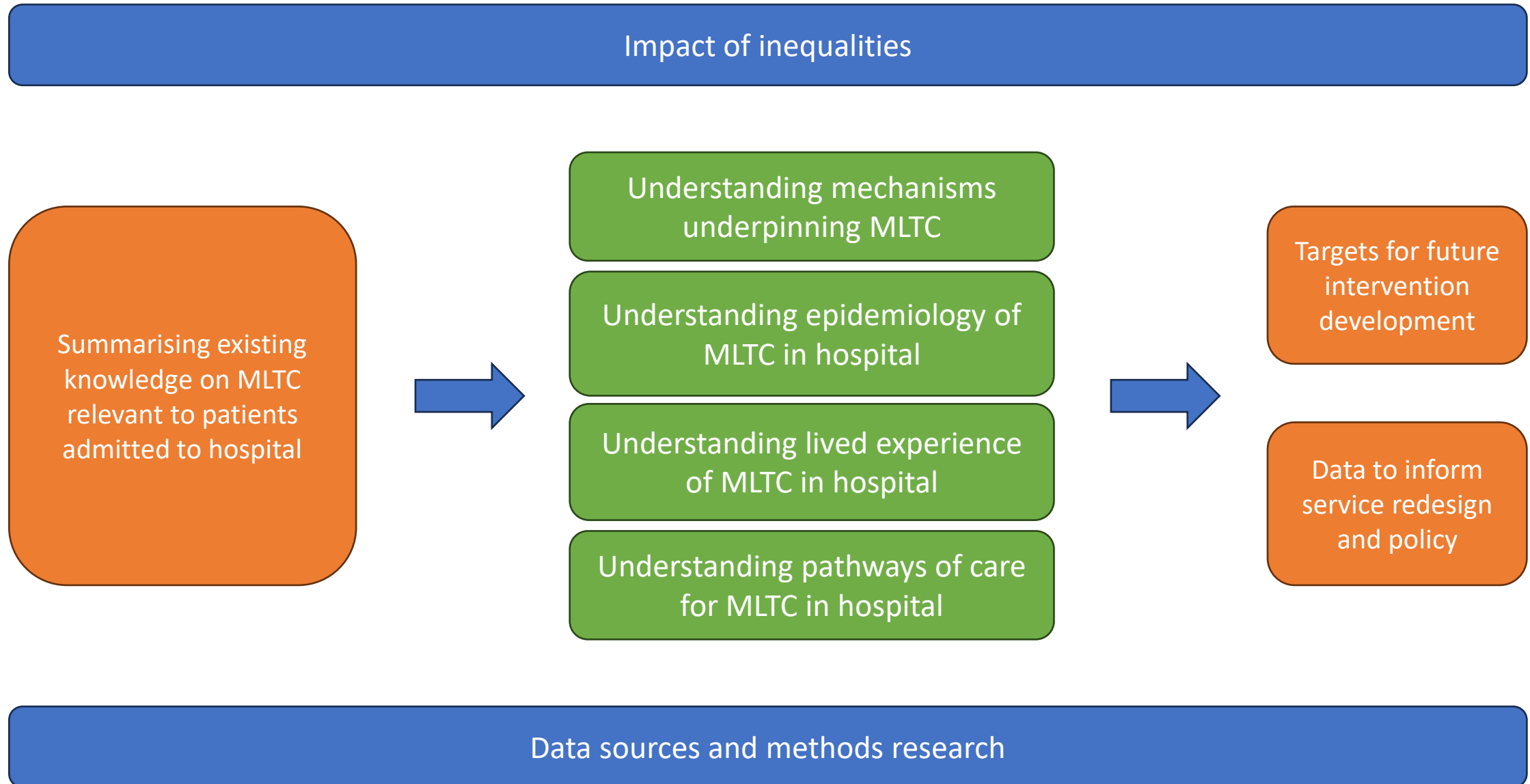

Supplement: Supplemental Material - Building ADMISSION – A research collaborative to transform understanding of multiple long-term conditions for people admitted to hospital [file sj-pdf-1-cob-10.1177_26335565251317940.pdf]
